# The role of diet in the pathogenesis and management of premature ovarian insufficiency

**DOI:** 10.3389/fnut.2026.1856944

**Published:** 2026-07-15

**Authors:** Liqi Pan, Yuxiao Yao, Wenlong Zhao, Xiangjuan Li

**Affiliations:** 1 Clinical Medical College, Hangzhou Normal University, Hangzhou, Zhejiang, China; 2 Hangzhou Women’s Hospital (Hangzhou Maternity and Child Health Care Hospital), Hangzhou, Zhejiang, China

**Keywords:** dietary patterns, inflammation, nutritional factors, ovarian reserve, oxidative stress, premature ovarian insufficiency

## Abstract

Premature ovarian insufficiency (POI) is characterized by an early decline in ovarian function before the age of 40 and is highly associated with infertility and long-term health issues. Increasing evidence suggests that dietary factors and nutritional status may influence ovarian aging and susceptibility to POI. Dietary patterns with high antioxidant and anti-inflammatory potential, such as the Mediterranean and plant-based diets, have been associated with more favorable indicators of ovarian reserve, whereas Western-style dietary patterns may accelerate ovarian functional decline. Nutrients, including vitamin D, omega-3 polyunsaturated fatty acids, and antioxidant compounds, may affect ovarian function through multiple biological processes, including modulation of oxidative stress, inflammation, mitochondrial activity, endocrine and metabolic homeostasis, epigenetic regulation, and the gut microbiota–ovarian axis. However, most available evidence is gained from observational studies and animal models. So far, the causal relationships have not yet been firmly established. Improvement in overall diet quality and appropriate supplementation of key nutrients may represent safe and potentially supportive strategies for the prevention and management of POI. Nevertheless, well-designed longitudinal studies and randomized controlled trials are required to clarify the effects of diet on ovary and to define optimal dietary intervention strategies for preserving ovarian health.

## Introduction

**Figure 1:**
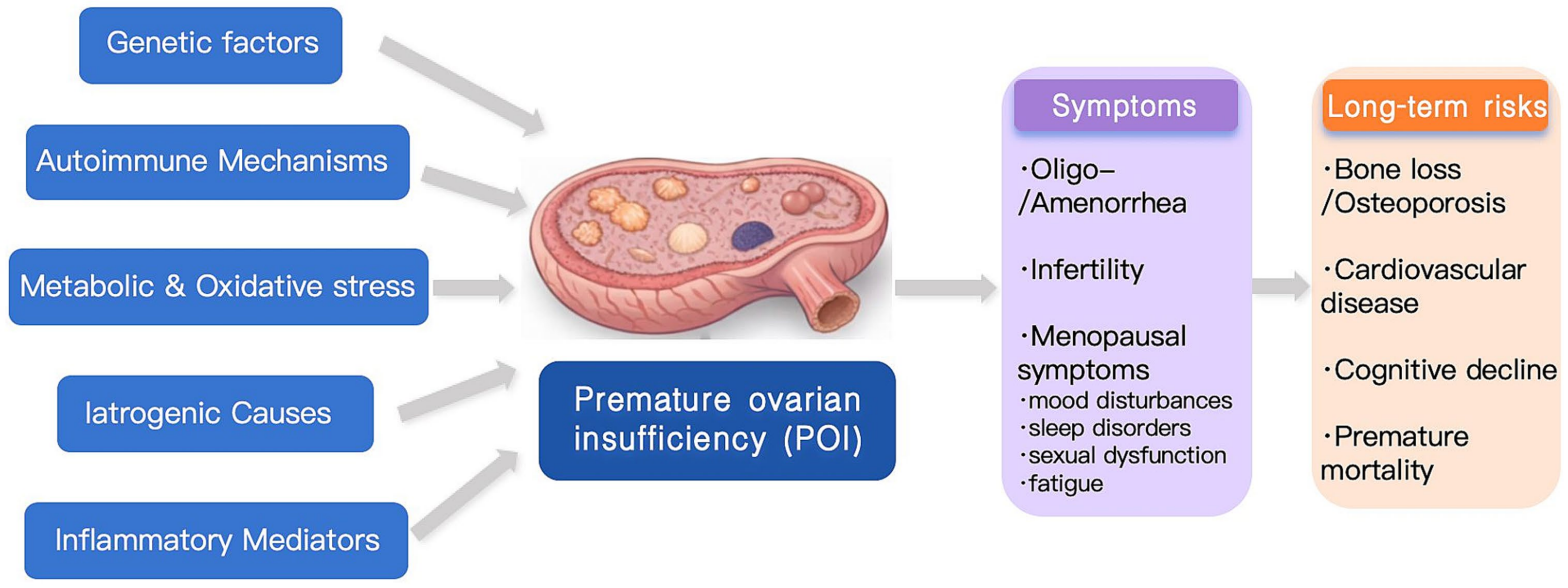
Pathogenesis and clinical manifestations of premature ovarian insufficiency (POI). Multiple etiological factors contribute to the development of POI, including genetic factors, autoimmune mechanisms, metabolic and oxidative stress, iatrogenic causes, and inflammatory mediators. These factors converge to impair ovarian function and accelerate follicular depletion. POI gives rise to a range of clinical symptoms, including oligo−/amenorrhea, infertility, and menopausal symptoms such as mood disturbances, sleep disorders, sexual dysfunction, and fatigue. Over time, the resulting estrogen deficiency predisposes affected individuals to several long-term health risks, including bone loss/osteoporosis, cardiovascular disease, cognitive decline, and premature mortality.

Premature ovarian insufficiency (POI) is defined as the depletion of ovarian follicles or the loss of follicular responsiveness to physiological gonadotropin stimulation in women younger than 40 years, resulting in premature menopause (1). The diagnostic criteria for POI include oligo/amenorrhea lasting for at least 4 months and elevated basal serum follicle-stimulating hormone (FSH) levels on two separate measurements obtained at least 4 weeks apart. Globally, POI affects approximately 4% of reproductive-age women (2, 3) and is associated with substantial adverse effects on physical, psychological, and reproductive health, ultimately impairing quality of life (Figure 1). Women with POI frequently experience depression and anxiety (4), as well as a range of menopausal manifestations, including mood disturbances, sleep disorders, sexual dysfunction, and fatigue (5). In addition, POI confers an increased long-term risk of multiple comorbidities, such as reduced bone mineral density (6), cognitive decline (7), cardiovascular disease (8), and premature mortality (9). Women with iatrogenic POI often present with an abrupt onset of symptoms that are typically more severe than those observed in natural menopause (10).

The pathogenesis of POI is highly complicated and involves the interactions among genetic, immunological, metabolic, environmental, and lifestyle-related factors. Genetic abnormalities represent one of the most clearly established causes of POI, with approximately 20–25% of affected women harboring identifiable defects, including chromosomal structural abnormalities, X chromosome deletions, or premutation of the *fragile X messenger ribonucleoprotein 1 (FMR1)* gene (11). Autoimmune-mediated ovarian damage constitutes another major etiological category, with approximately 5–10% of patients presenting with concomitant autoimmune disorders (12). Persistent elevation of inflammatory mediators, such as interleukin-6 (IL-6), tumor necrosis factor-*α* (TNF-α), and C-reactive protein (CRP), may accelerate follicular apoptosis and stromal fibrosis, ultimately leading to depletion of the ovarian reserve. Metabolic dysregulation and oxidative stress have also been implicated in the development of POI. Insulin resistance, dyslipidemia, and obesity may impair mitochondrial function in ovarian granulosa cells by increasing the production of reactive oxygen species (ROS) and promoting lipid peroxidation, thereby contributing to follicular loss (13). In addition, iatrogenic injuries resulting from chemotherapy, radiotherapy, pelvic irradiation, or surgical interventions represent important causes of POI. Environmental endocrine-disrupting chemicals have also been suggested to reduce ovarian reserve through estrogen receptor–mediated pathways, oxidative stress, and epigenetic modifications.

Recently, lifestyle-related factors, including dietary patterns, smoking, alcohol consumption, sleep deprivation, and psychological stress, have been increasingly recognized as potential modulators of ovarian function (14). Among these, diet has attracted growing attention as a modifiable environmental exposure. Dietary intake may influence ovarian physiology through multiple biological processes, including regulation of energy metabolism, oxidative stress, inflammatory responses, and steroid hormone synthesis.

Evidence from Western populations indicates that greater adherence to the Mediterranean diet is associated with improved reproductive outcomes and more favorable markers of ovarian function (15, 16). Antioxidant nutrients, such as vitamins C and E and polyphenolic compounds, as well as omega-3 polyunsaturated fatty acids, may exert protective effects on ovary, whereas high intake of refined sugars, trans fatty acids, and red meat has been linked to an increased risk of ovarian dysfunction (17, 18). However, the investigations specifically focusing on women with POI remain limited, and almost all existing evidence is obtained from studies conducted in the context of infertility, polycystic ovary syndrome (PCOS), or natural menopause. Moreover, most published studies are limited by relatively small sample sizes, and integrated analyses of dietary patterns with clinical and molecular markers of ovarian function remain scarce.

In China, several studies have performed the associations of vitamin D status, trace elements, dietary structure, and ovarian reserve (19). However, robust evidence is still lacking, and the role of dietary factors in the development of POI has not been clearly established. In addition, Chinese women show distinct dietary patterns, characterized by relatively high carbohydrate intake, predominant use of plant-based oils, and low dietary fiber intake, which may have differential effects on ovarian functions compared with Western populations.

Therefore, investigating the role of diet in the pathogenesis of POI is of substantial scientific and clinical relevance. These researches may help elucidate potential links between dietary patterns and ovarian aging and may provide novel perspectives for dietary-based prevention and management strategies. Furthermore, it may facilitate the development of evidence-based nutritional guidance and public health interventions aimed at improving female reproductive health and quality of life.

This review aims to comprehensively summarize the evidence from 2000 to 2026, regarding the influence of diet on POI from the perspectives of pathogenic mechanisms, dietary patterns, nutrient-related biological pathways, epidemiological findings, and clinical intervention strategies, with the objective of providing practical dietary insights for clinical practice and identifying key directions for future research.

Table 1 provides a structured overview of key dietary factors discussed in the review, proposed mechanisms, and clinical implications.

**Table 1:** Summary of key dietary factors in the pathogenesis and management of premature ovarian insufficiency: proposed mechanisms and clinical implications.

Dietary factor	Proposed mechanisms	Clinical implications
Dietary patterns		
Mediterranean diet	Reduced systemic inflammation and oxidative stress; improved insulin sensitivity; maintenance of gut microbiota homeostasis; enhanced antioxidant capacity	Greater adherence associated with higher AMH levels, lower FSH concentrations, and slower decline in ovarian reserve
Western dietary pattern	Aggravated oxidative stress and inflammation; promoted insulin resistance; accelerated follicular depletion through mitochondrial dysfunction and NF-κB activation; altered gut microbiota	Associated with lower AMH levels and higher risk of diminished ovarian reserve; long-term consumption of ultra-processed foods may increase susceptibility to POI
Plant-based and anti-inflammatory dietary patterns	Lower pro-inflammatory dietary load; higher intake of phytochemicals and dietary fiber; higher plant-derived protein intake	Higher plant protein intake associated with more favorable markers of ovarian reserve; higher DII scores associated with lower AMH and increased risk of diminished ovarian reserve
High-fat diets and obesity-related dietary patterns	Directly aggravates oxidative stress, inflammation, and mitochondrial injury in ovarian tissue; alters adipokine secretion including leptin and adiponectin	Animal models demonstrate accelerated follicular atresia; high-fat diets may indirectly increase risk of POI through metabolic and endocrine dysregulation
High dietary acid load	Elevated PRAL associated with low-grade metabolic acidosis, inflammation, and altered hormone metabolism	Elevated dietary acid load associated with increased risk of diminished ovarian reserve
Specific nutrients		
Vitamin D	Regulates AMH and FSH expression via vitamin D receptor (VDR) activation in oocytes and granulosa cells	Vitamin D deficiency associated with lower AMH; meta-analytic evidence shows weak or population-specific associations; effects of supplementation remain inconclusive
Omega-3 PUFAs (EPA/DHA)	Inhibits NF-κB signaling; reduces expression of inflammatory mediators; improves mitochondrial function in granulosa cells; reduces follicular atresia in animal models	Higher dietary intake of ω-3 fatty acids associated with lower FSH; ω-3 supplementation may reduce serum FSH; evidence for direct effect on AMH remains limited
Antioxidant nutrients	Scavenge ROS; preserve mitochondrial function; maintain genomic stability in oocytes and granulosa cells; resveratrol modulates sirtuin signaling and promotes mitochondrial biogenesis	Higher dietary antioxidant capacity associated with higher AMH and lower risk of diminished ovarian reserve; resveratrol improves oocyte quality and ameliorates epigenetic alterations in ovarian tissue
BCAAs	BCAA insufficiency activates ceramide–ROS axis, inducing mitochondrial damage and contributing to ovarian dysfunction	Animal studies demonstrate BCAA insufficiency leads to a POI-like phenotype; population-based evidence in humans remains limited
Emerging dietary strategies		
Caloric restriction (CR)	Upregulates SIRT1, SIRT6, FOXO3a, and NRF1; suppresses mTORC1 activity; reduces premature follicle activation, granulosa cell apoptosis; enhances antioxidant capacity	30% CR (3–11 months) preserved primordial follicle numbers in mice; moderate CR attenuated ovarian fibrosis in rhesus macaques; effects are duration-dependent; human evidence lacking
Intermittent fasting (IF)	Enhances autophagy; improves insulin sensitivity; reduces circulating TNF-α and IL-6; favorably modulates adipokine secretion	IF may suppress HPO axis and reduce AMH/FSH/estradiol in lean women; more beneficial in women with metabolic dysfunction (PCOS); safety in women at risk of POI not established
Dietary endocrine-disrupting contaminants		
Bisphenol A (BPA)	Binds ERα/ERβ as xenoestrogen; impairs steroidogenesis; promotes granulosa cell apoptosis; accelerates primordial follicle activation and depletion; induces epigenetic alterations in ovarian tissue	Linked to reduced AFC and lower AMH; epigenetic changes may persist across generations; minimize exposure from food-contact plastics and can linings
PFAS (PFOA, PFOS)	Induces oxidative stress and ovarian cell apoptosis; disrupts granulosa cell–oocyte communication; impairs steroidogenesis; interferes with epigenetic regulation and hypothalamic kisspeptin signaling	Higher plasma PFOA/PFOS/PFHxS associated with increased POI risk; detectable in follicular fluid

AFC, antral follicle count; AMH, anti-Müllerian hormone; AMPK, AMP-activated protein kinase; BCA As, branched-chain amino acids; BPA, bisphenol A; CRP, C-reactive protein; DHA, docosahexaenoic acid; DII, dietary inflammatory index; EPA, eicosapentaenoic acid; FOXO3a, forkhead box O3a; HEI, healthy eating index; HPO, hypothalamic–pituitary–ovarian; IF, intermittent fasting; IL-6, interleukin-6; mTORC1, mammalian target of rapamycin complex 1; NF-κB, nuclear factor kappa B; PFAS, per- and polyfluoroalkyl substances; PFOA, perfluorooctanoic acid; PFOS, perfluorooctane sulfonic acid; PGC-1α, peroxisome proliferator-activated receptor-gamma coactivator 1-alpha; POI, premature ovarian insufficiency; PCOS, polycystic ovary syndrome; PRAL, potential renal acid load; PUFAs, polyunsaturated fatty acids; ROS, reactive oxygen species; SIRT1, sirtuin 1; TRE, time-restricted eating; TNF-α, tumor necrosis factor-alpha; VDR, vitamin D receptor.

## Methodology

Literature relevant to diet and POI was searched in PubMed/MEDLINE, Web of Science, Embase, Scopus, and China National Knowledge Infrastructure (CNKI). Studies published between January 2000 to March 2026, without language restrictions. Reference lists of selected articles and relevant reviews were also examined to identify additional studies.

Key search terms included combinations of terms related to:

(1) POI and ovarian aging (“premature ovarian insufficiency,” “premature ovarian failure,” “diminished ovarian reserve,” “ovarian aging,” “early menopause”);
(2) Dietary patterns and nutritional factors (“diet,” “Mediterranean diet,” “Western diet,” “vitamin D,” “omega-3 fatty acids,” “antioxidants,” “gut microbiota”);
(3) Metabolic and mechanistic pathways (“oxidative stress,” “inflammation,” “mitochondrial function,” “epigenetic regulation,” “SIRT1,” “mTOR,” “autophagy”); and.
(4) Dietary endocrine-disrupting contaminants (“bisphenol A,” “PFAS,” and “reproductive toxicity”).

Studies were included if they: (1) investigated associations between dietary patterns, specific nutrients, nutritional interventions, caloric or intermittent fasting strategies, or dietary contaminant exposures and ovarian function, ovarian reserve, or POI; (2) involved human subjects of reproductive age or included *in vitro* or animal models relevant to ovarian biology; and (3) were original research articles, systematic reviews, meta-analyses, or randomized controlled trials. Studies were excluded if they were conference abstracts, editorials, letters, or case reports without quantitative data, or if the primary outcome was unrelated to ovarian function or reproductive health. Because relatively few studies have focused specifically on POI, evidence from related conditions, including diminished ovarian reserve, infertility, PCOS, and natural menopause, was also included when relevant to the mechanistic or clinical themes discussed in this review. For the sections on caloric restriction, intermittent fasting, and dietary endocrine-disrupting contaminants where human POI data remain limited—mechanistic evidence from animal models and epidemiological data from general reproductive-age populations were also included to provide mechanistic context.

## General etiology of POI and the role of diet in POI

### Oxidative stress and impaired antioxidant defense

Oxidative stress is considered a major contributor in the pathogenesis of POI (20). Oocytes and surrounding granulosa cells are vulnerable to redox imbalance during folliculogenesis, steroidogenesis, and ovulation (21). Excessive accumulation of reactive oxygen species (ROS), together with impaired antioxidant defenses, leads to lipid peroxidation, protein oxidation, and DNA damage, ultimately leading to follicular atresia and oocyte apoptosis (22).

Diet is an important determinant of oxidative stress at both the systemic and ovarian levels. Several observational studies and systematic reviews have shown that diets rich in antioxidant nutrients, including vitamins C and E, carotenoids, and polyphenolic compounds, are associated with higher anti-Müllerian hormone (AMH) levels and a slower decline in ovarian reserve (23, 24). In contrast, dietary patterns characterized by high intake of saturated fat, refined carbohdyrates, and ultra-processed foods increase systemic oxidative burden, and may promote depletion of the follicular pool. However, the majority of evidence linking dietary antioxidant intake to ovarian reserve derives from cross-sectional observational studies using self-reported dietary assessment tools, which are subject to substantial recall bias and confounding by overall lifestyle. Prospective studies with objective biomarkers of oxidative stress and ovarian reserve are needed to confirm whether dietary antioxidant modification translates into meaningful clinical benefit in women at risk of POI.

### Mitochondrial dysfunction and altered energy metabolism

The oocyte is a mitochondria-rich cell. Intact mitochondrial function is essential for oocyte maturation, fertilization, and early embryonic development. Accumulating evidence indicates that mitochondrial DNA (mtDNA) mutations, reduced mtDNA copy number, and impaired oxidative phosphorylation efficiency are key pathological features associated with ovarian functional decline (25, 26).

Nutritional status may influence ovarian function in part through effects on mitochondrial metabolism and cellular energy metabolism. Several dietary components, such as omega-3 polyunsaturated fatty acids, coenzyme Q10 (27), and resveratrol (28), can activate the AMP-activated protein kinase (AMPK)–peroxisome proliferator-activated receptor gamma coactivator-1α (PGC-1α) signaling pathway, thereby enhancing mitochondrial activity and improving oocyte quality. In contrast, insufficient intake of branched-chain amino acids (BCAAs) has been associated with mitochondrial stress via activation of the ceramide–ROS axis, ultimately contributing to ovarian dysfunction in experimental models (29).

### Inflammation and immune dysregulation

Chronic low-grade inflammation is increasingly recognized as an important contributor to ovarian aging (30). Elevated concentrations of proinflammatory mediators, including tumor necrosis factor-*α* (TNF-α), interleukin-6 (IL-6), and C-reactive protein (CRP), can suppress follicular development and promote granulosa cell apoptosis through activation of inflammatory pathways such as nuclear factor-κB (NF-κB) (31) signaling pathway.

Dietary patterns are important regulators of systemic and local inflammatory status. Anti-inflammatory dietary patterns, exemplified by the Mediterranean diet, have been associated with reduced circulating inflammatory markers and more favorable indicators of ovarian reserve (32). In contrast, high consumption of saturated fat and refined carbohydrates has been shown to promote ovarian inflammatory responses and accelerate follicular loss. Moreover, autoimmune mechanisms contribute substantially to POI in a subset of patients, and dietary factors may indirectly influence the risk of autoimmune-related POI by modulating immune tolerance and inflammatory pathways (33).

### Endocrine and metabolic dysregulation

Ovarian function is tightly regulated by the hypothalamic–pituitary–ovarian (HPO) axis and is sensitive to metabolic disturbances, including insulin resistance (34), obesity, and metabolic syndrome (35). These metabolic abnormalities may disrupt ovarian steroidogenesis and follicular development through alterations in sex hormone–binding globulin (SHBG) concentrations, gonadotropin secretion, and peripheral hormone metabolism.

Diet strongly influences insulin sensitivity and metabolic homeostasis. Diets with a high glycemic load worsen insulin resistance and enhance oxidative and inflammatory stress within ovarian tissue (36), whereas dietary patterns characterized by high fiber intake and low glycemic index contribute to improved metabolic profiles and endocrine balance (19). Vitamin D, a secosteroid hormone, has also been implicated in ovarian function through regulation of the expression of AMH and FSH via activation of the vitamin D receptor (VDR). Although finds remain inconsistent across populations, several studies suggest a potential role in ovarian endocrine function (37).

### Epigenetic regulation and ovarian aging

In addition to genetic factors, ovarian aging is influenced by epigenetic mechanisms, including DNA methylation, histone modifications, and non-coding RNAs (38, 39). Epigenetic alterations in oocytes and granulosa cells may disrupt the expression of genes involved in follicle recruitment, growth, and apoptosis, potentially contributing to accelerated ovarian aging and follicular loss (40).

Nutritional factors can influence epigenetic regulation. Nutrients involved in one-carbon metabolism, such as folate, vitamin B12, and choline, influence DNA methylation patterns in ovarian cells (41). In addition, polyphenolic compounds, including resveratrol, have been reported to modulate sirtuin signaling pathways, enhance mitochondrial function, and may contribute to delayed ovarian aging through epigenetic and metabolic effects (42).

### The gut microbiota–ovary axis

Emerging evidence indicates that the gut microbiota serves as a key intermediary linking diet and ovarian function (43). Gut microbial dysbiosis may influence ovarian physiology by alterations in estrogen metabolism, inflammatory signaling, and metabolic homeostasis (44). Microbial metabolites, particularly short-chain fatty acids (SCFAs), have anti-inflammatory and antioxidant properties and may help maintain the ovarian microenvironment (45).

Diet strongly shapes gut microbial composition. Diets rich in dietary fiber, probiotics, and fermented foods are associated with greater microbial diversity and metabolic health, whereas high-fat diets and ultra-processed foods induce dysbiosis and promote dysbiosis and chronic inflammation, potentially contributing to ovarian aging and functional decline (46). However, most human studies in this area are cross-sectional, involve small sample sizes, and have been conducted primarily in women with PCOS or infertility rather than with POI specifically. Whether gut microbiota modification mediates the effects of diet on ovarian reserve has not been established, and longitudinal dietary intervention studies with concurrent microbiome and ovarian reserve measurements are needed.

**Figure 2:**
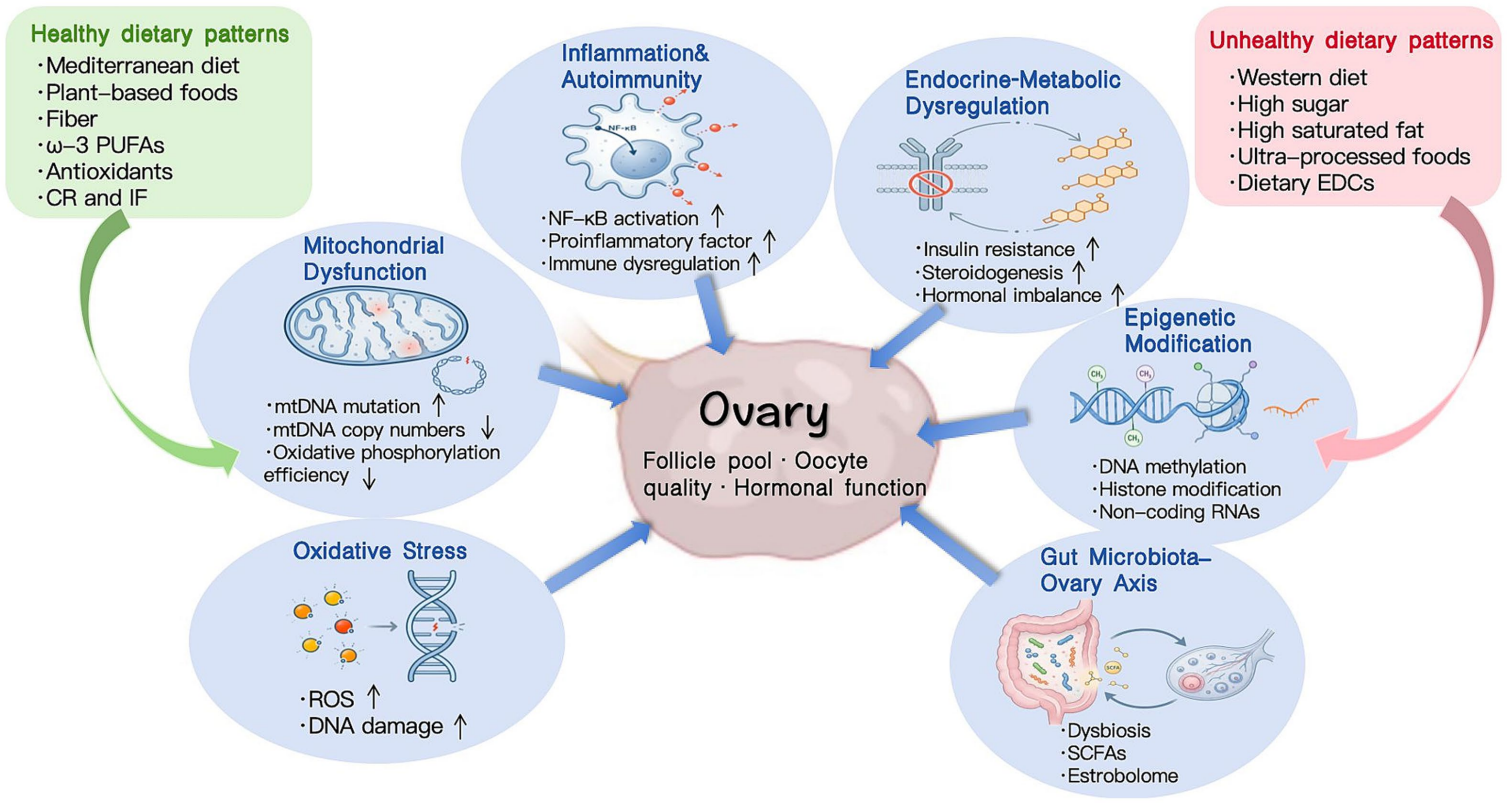
Proposed mechanisms linking dietary factors to the pathogenesis of POI. Healthy dietary patterns—including the Mediterranean diet, plant-based foods, fiber, ω-3 PUFAs, antioxidants, and CR and IF—exert protective effects on the ovary, whereas unhealthy dietary patterns —including the Western diet, high sugar, high saturated fat, and ultra-processed foods—exert detrimental effects. Of note, dietary EDCs can be ingested through the diet and represent an emerging dietary risk factor contributing to POI. These dietary inputs converge on the ovary, influencing the follicle pool, oocyte quality, and hormonal function through six interconnected mechanisms: mitochondrial dysfunction, inflammation and autoimmunity, endocrine-metabolic dysregulation, epigenetic modification, the gut microbiota–ovary axis, and oxidative stress. Arrows (↑/↓) denote the direction of change. Abbreviations: CR, caloric restriction; EDCs, endocrine-disrupting chemicals; IF, intermittent fasting; mtDNA, mitochondrial DNA; NF-κB, nuclear factor kappa B; POI, premature ovarian insufficiency; PUFAs, polyunsaturated fatty acids; ROS, reactive oxygen species; SCFAs, short-chain fatty acids.

The major biological pathways connecting diet and POI are summarized in Figure 2. POI develops through multiple overlapping biological processes, including oxidative stress, mitochondrial dysfunction, inflammatory and immune dysregulation, endocrine and metabolic disturbances, epigenetic alterations, and gut microbiota dysbiosis. Dietary factors may influence ovarian function through these pathways, thereby supporting the rationale for nutritional strategies in the prevention and management of POI.

## Dietary patterns and the risk of POI

Compared with examining individual nutrients, dietary pattern analysis better reflects long-term dietary habits and accounts for interactions among multiple dietary components. Growing evidence from epidemiological studies and systematic reviews suggests associations between overall dietary patterns, ovarian reserve decline, and the risk of POI (47, 48).

### Mediterranean diet

The Mediterranean diet is characterized by high consumption of vegetables, fruits, whole grains, legumes, nuts, olive oil, and fish, along with a low intake of red meat, processed foods, and refined sugars. It has been consistently associated with anti-inflammatory and antioxidant effects (49).

Greater adherence to the Mediterranean diet has been associated with higher serum anti-Müllerian hormone (AMH) levels, lower follicle-stimulating hormone (FSH) concentrations, and a slower decline in ovarian reserve (50). Proposed mechanisms include reduced systemic inflammation, improved insulin sensitivity, enhanced antioxidant capacity, and modulation of gut microbiota composition. A recent systematic review further suggested that the Mediterranean diet may protect ovarian function through coordinated effects on multiple biological pathways. However, most current evidence remains observational, high-quality prospective studies are needed to confirm these findings and to determine whether these associations extend to POI risk (15).

### Western dietary pattern

The Western dietary pattern generally includes high intake of saturated and *trans* fats, refined carbohydrates, low dietary fiber, and high energy density, and has been linked to metabolic dysfunction and chronic low-grade inflammation (51).

Adherence to a Western dietary pattern has been associated with lower AMH levels and a higher risk of diminished ovarian reserve (18). High-fat and high-sugar diets may increase oxidative stress and inflammation, promote insulin resistance, and accelerate follicular depletion through mitochondrial dysfunction and activation of inflammatory signaling pathways. In addition, long-term consumption of ultra-processed foods may also influence estrogen metabolism and ovarian function through alterations in gut microbiota composition, potentially contributing to POI risk (44).

### Plant-based and anti-inflammatory dietary patterns

Plant-based dietary patterns are typically rich in dietary fiber, vitamins, minerals, and phytochemicals with antioxidant and anti-inflammatory properties. Several studies suggest that a higher proportion of plant-derived protein intake is associated with more favorable ovarian reserve markers (52), whereas excessive consumption of animal-derived protein may adversely affect ovarian function.

The Dietary Inflammatory Index (DII) has been used to assess the inflammatory potential of diet. Higher DII scores, reflecting more pro-inflammatory dietary patterns, have been associated with lower AMH levels and an increased risk of diminished ovarian reserve (53). These findings further support the potential role of chronic low-grade inflammation as a key mechanism linking dietary exposure to ovarian aging and POI development.

### Asian and traditional Chinese dietary patterns

Dietary patterns in Asian populations, particularly among Chinese women, differ substantially from Western populations. These dietary patterns are frequently characterized by a relatively high carbohydrate intake, the predominant use of plant-based oils, and a generally lower intake of dietary fiber. These dietary characteristics may have complex effects on ovarian function. For instance, a high carbohydrate consumption, particularly if it involves a high glycemic load, can exacerbate insulin resistance and increase oxidative and inflammatory stress within ovarian tissue. Furthermore, inadequate dietary fiber intake may limit some of the metabolic benefits associated with high-fiber diets, which are known to contribute to improved metabolic profiles. Although several studies in China have begun to investigate the associations among dietary structure, trace elements, vitamin D status, and ovarian reserve, direct evidence linking the role of these specific dietary factors in the development of POI is still lacking. Consequently, defining how the traditional Chinese dietary pattern influences ovarian aging remains a critical area of substantial scientific and clinical relevance.

### High-fat diets and obesity-related dietary patterns

High-fat diets and chronic excess energy intake contribute to obesity and metabolic syndrome, both of which are recognized risk factors for ovarian dysfunction (17). Experimental studies in animal models have demonstrated that high-fat diets directly increase oxidative stress, inflammation, and mitochondrial dysfunction in ovarian tissue (54).

In population-based studies, the relationship between body mass index (BMI) and ovarian reserve markers did not establish well. However, several investigations suggest that obesity-related dietary patterns may indirectly increase the risk of POI through metabolic and endocrine dysregulation. Moreover, high-fat diets may further affect hypothalamic–pituitary–ovarian (HPO) axis activity by altering adipokine secretion, including leptin and adiponectin, from adipose tissue (55).

### Dietary acid load and beverage consumption patterns

Dietary acid load has recently gained attention as a marker of overall dietary composition. Diets high in animal protein and low in fruits and vegetables generally have a higher potential renal acid load (PRAL). Case–control studies have shown that elevated dietary acid load is associated with an increased risk of diminished ovarian reserve, possibly through low-grade metabolic acidosis, inflammation, and altered hormone metabolism (56).

Beverage consumption patterns may also influence ovarian function. High intake of sugar-sweetened beverages and caffeine has been associated with impaired ovarian reserve, possibly by exacerbating insulin resistance and oxidative stress. In contrast, healthier beverage patterns characterized by higher consumption of water, unsweetened tea, and low-sugar beverages appear to be associated with a lower risk of ovarian reserve impairment (57).

### Overall dietary quality indices

Composite dietary quality scores, such as the Healthy Eating Index (HEI), have been widely used to assess overall adherence to dietary guidelines. Evidence suggests that higher HEI scores are associated with higher AMH levels and a reduced risk of diminished ovarian reserve (58). These findings suggest that overall dietary quality, rather than the intake of single nutrients, may influence ovarian aging and POI risk.

Collectively, current evidence indicates that distinct dietary patterns exert differential effects on ovarian reserve and POI risk. High-quality dietary patterns characterized by anti-inflammatory and antioxidant properties, including the Mediterranean diet and plant-based diets, appear to be associated with more favorable ovarian reserve indicators. In contrast, Western dietary patterns high in saturated fats, refined carbohydrates, and ultra-processed foods are linked to an increased risk of POI. However, most available data are observational in nature, and well-designed prospective cohort studies and randomized controlled trials are required to clarify causal relationships and to identify optimal dietary strategies for preserving ovarian function.

## Specific nutrients and POI

In addition to overall dietary patterns, increasing attention has been directed toward the role of specific nutrients in ovarian function and POI development. Current evidence has primarily focused on vitamin D, omega-3 polyunsaturated fatty acids, antioxidant nutrients, amino acid metabolism, and selected micronutrients.

### Vitamin D

Vitamin D is a secosteroid hormone that regulates gene transcription through activation of the vitamin D receptor (VDR). The presence of VDR in ovarian tissue, including oocytes and granulosa cells, provides a biological basis for the involvement of vitamin D in ovarian physiology.

Several observational studies and meta-analyses have evaluated associations between circulating 25-hydroxyvitamin D concentrations and markers of ovarian reserve, including anti-Müllerian hormone (AMH), follicle-stimulating hormone (FSH), and antral follicle count (AFC) (37). Several studies have reported that vitamin D deficiency is associated with lower AMH levels, suggesting a potential role in the maintenance of ovarian reserve (59). However, large cohort studies have failed to demonstrate consistent associations between vitamin D status and ovarian reserve (60).

Overall, meta-analytic evidence suggests that vitamin D shows weak or population-specific associations with AMH, and these relationships may vary according to age, ethnicity, body mass index (BMI), and baseline reproductive status. Similarly intervention studies evaluating vitamin D supplementation on ovarian reserve remain inconclusive. Intervention studies have been predominantly conducted in infertile women undergoing assisted reproduction rather than in women with established POI, and most trials were small, short-term, and lacked placebo controls. Current evidence is therefore insufficient to support vitamin D supplementation as a targeted strategy for POI beyond the correction of documented deficiency, and adequately powered, POI-specific trials are required (61, 62).

### Omega-3 polyunsaturated fatty acids

Omega-3 polyunsaturated fatty acids (ω-3 PUFAs), primarily eicosapentaenoic acid (EPA) and docosahexaenoic acid (DHA), exhibit anti-inflammatory and antioxidant properties and influence membrane fluidity and mitochondrial function (63).

Studies in ovarian granulosa cells have shown that *ω*-3 PUFAs can inhibit nuclear factor-κB (NF-κB) signaling, reduce the expression of inflammatory mediators, and improve mitochondrial function (64). In mouse models, *ω*-3 PUFA supplementation has been reported to reduce follicular atresia, improve oocyte quality, and delay ovarian aging (65).

In human observational studies, higher dietary intake or circulating levels of *ω*-3 fatty acids have been associated with lower FSH concentrations and more favorable reproductive endocrine profiles (66). One prospective intervention study suggested that *ω*-3 PUFA supplementation may reduce serum FSH levels in women of reproductive age, although evidence for a direct effect on AMH remains limited (67). Overall, the available evidence suggests that ω-3 PUFAs may support ovarian function indirectly by improving the ovarian microenvironment and attenuating systemic inflammation; however, mechanistic data derive predominantly from *in vitro* and animal models at doses that often exceed those achievable through dietary intake, and available human studies have been conducted primarily in IVF populations rather than women with POI. Large-scale, placebo-controlled trials with prespecified ovarian reserve endpoints in a POI-specific population are therefore needed before supplementation can be incorporated into evidence-based clinical guidelines (68).

### Antioxidant nutrients

Given the central role of oxidative stress in the pathogenesis of POI, antioxidant nutrients have been extensively investigated. These include vitamin C (69), vitamin E (70), β-carotene (71), selenium (70), zinc (72), and polyphenolic compounds (71).

Several observational studies have reported that higher dietary antioxidant capacity is associated with higher AMH levels and a lower risk of diminished ovarian reserve (73). Antioxidants may mitigate follicular depletion by scavenging reactive oxygen species, preserving mitochondrial function, and maintaining genomic stability in oocytes and granulosa cells (24).

Resveratrol, a naturally occurring polyphenol, has received particular attention because of its ability to activate sirtuin signaling pathways and promote mitochondrial biogenesis. *In vitro* studies indicate that resveratrol improves oocyte quality, delays ovarian aging (28), and ameliorates epigenetic alterations in ovarian tissue (42). However, high-quality clinical evidence regarding its long-term effects on ovarian reserve in humans remains limited. More broadly, associations between dietary antioxidant intake and ovarian reserve are difficult to disentangle from the effects of overall dietary quality, as antioxidant-rich diets also tend to be anti-inflammatory and nutrient-dense in multiple other respects. Randomized controlled trials of individual antioxidant supplementation in women with POI remain scarce.

### Branched-chain amino acids and protein intake patterns

Branched-chain amino acids (BCAAs), including leucine, isoleucine, and valine, play important roles in cellular energy metabolism and mitochondrial homeostasis. Recent animal studies have demonstrated that BCAA insufficiency can induce ovarian dysfunction and a POI-like phenotype through activation of the ceramide–reactive oxygen species axis (29).

In cohort studies, the source of dietary protein appears to be more relevant than total protein intake. Higher consumption of plant-derived protein has been associated with more favorable markers of ovarian reserve (53), whereas excessive intake of animal-derived protein may be linked to accelerated ovarian functional decline. Nevertheless, population-based evidence specifically addressing the relationship between BCAAs and POI remains limited, and further studies are required to define optimal intake ranges and clinical relevance.

### One-carbon metabolism–related nutrients and trace elements

Nutrients involved in one-carbon metabolism, including folate, vitamin B12, and choline, are essential for DNA synthesis and methylation (74). Deficiencies in these nutrients may lead to epigenetic dysregulation, potentially affecting follicular development and ovarian function (40).

Trace elements such as selenium and zinc are integral components of endogenous antioxidant enzyme systems and play a critical role in maintaining ovarian redox homeostasis. Although some observational studies have suggested associations between inadequate trace element intake and impaired ovarian function, existing evidence is largely derived from small-scale studies and remains inconclusive (75).

Collectively, current evidence suggests that vitamin D, omega-3 polyunsaturated fatty acids, antioxidant nutrients, amino acids, and selected micronutrients may influence ovarian function through multiple biological pathways, including modulation of oxidative stress, inflammation, mitochondrial activity, and epigenetic regulation. However, the strength of evidence varies considerably among nutrients, with most data derived from observational studies or experimental models. Well-designed randomized controlled trials specifically targeting women with POI or diminished ovarian reserve are still lacking. Future research should prioritize evaluation of dose–response relationships, synergistic interactions among nutrients, and the development of personalized nutritional intervention strategies.

## Clinical implications of dietary interventions in the prevention and management of POI

Although the etiology of POI is complex and remains incompletely understood, accumulating evidence suggests that diet, as a modifiable and low-risk lifestyle factor, may have clinical relevance in primary prevention, in delaying disease progression, and in supporting comprehensive management strategies.

### Role of dietary interventions in the primary prevention of POI

For women who have not yet developed overt ovarian failure but present with established risk factors, such as a family history of POI, autoimmune disorders, metabolic abnormalities, or unfavorable lifestyle behaviors, appropriate dietary modification may contribute to risk reduction.

Epidemiological evidence indicates that habitual adherence to high-quality dietary patterns, including the Mediterranean diet and other anti-inflammatory dietary profiles, is associated with a slower decline in ovarian reserve. These patterns, characterized by high intake of antioxidants, omega-3 polyunsaturated fatty acids, and dietary fiber, may exert protective effects on ovarian function by alleviating oxidative stress, improving inflammatory status, and maintaining metabolic homeostasis. Accordingly, promotion of healthy dietary habits among women of reproductive age, particularly those at increased risk, may represent a feasible prevention strategy with potential public health significance.

### Potential role of dietary interventions in early ovarian dysfunction

In women who exhibit early signs of declining ovarian reserve but have not yet progressed to established POI, dietary interventions may help slow disease progression. Several studies have suggested that improvements in overall diet quality are associated with a reduced rate of decline in AMH concentrations during the early stages of ovarian dysfunction.

The potential benefits of nutritional intervention may be mediated through multiple mechanisms, including attenuation of systemic inflammation, enhancement of mitochondrial function, preservation of endocrine balance, and reinforcement of antioxidant defenses. Although randomized controlled trials specifically designed to evaluate the efficacy of dietary modification in delaying the onset of POI are currently lacking, these observational findings provide a theoretical basis for considering dietary counseling as part of early-stage management.

### Role of dietary interventions in comprehensive POI management

In women with established POI, hormone replacement therapy (HRT) remains the cornerstone of treatment for alleviating symptoms related to hypoestrogenism and for preventing long-term complications such as osteoporosis and cardiovascular disease. Nevertheless, dietary interventions may serve as an important adjunct to standard therapy by contributing to overall metabolic health and by reducing the burden of comorbid conditions.

Appropriate dietary patterns may help mitigate the metabolic disturbances and chronic inflammatory state frequently observed in women with POI. Diets rich in calcium, vitamin D, and high-quality protein are particularly relevant for skeletal health, whereas dietary patterns abundant in omega-3 fatty acids and antioxidant nutrients may contribute to cardiovascular risk reduction. In addition, dietary modulation of the gut microbiota has been proposed as a potential mechanism through which nutrition may influence psychological well-being and overall quality of life in this population (76).

### Individualized nutritional interventions and precision medicine

Given the pronounced heterogeneity in both the etiology and clinical presentation of POI, uniform dietary recommendations are unlikely to be optimal for all patients. The emerging concept of precision nutrition emphasizes the integration of genetic background, metabolic characteristics, gut microbiota composition, and lifestyle factors to guide individualized dietary interventions (77).

Future research may explore stratified nutritional strategies based on genetic polymorphisms, metabolomic profiles, or microbial signatures, with the aim of improving the efficacy and reproducibility of dietary interventions in the prevention and management of POI. Furthermore, multimodal management approaches combining dietary modification with physical activity, psychological support, and pharmacological therapy may better reflect the multifactorial nature of POI and improve patient-centered outcomes.

### Limitations of current evidence and considerations for clinical practice

Despite increasing interest in dietary approaches for POI management, the current body of evidence has several important limitations. Most studies are observational in design, which restricts causal inference. In addition, variability in the diagnostic criteria for POI and early ovarian dysfunction contributes to heterogeneity across studies. Dietary intake is commonly assessed using self-reported questionnaires, which are subject to recall bias and measurement error.

Therefore, in clinical practice, dietary interventions should be regarded as supportive and complementary strategies rather than substitutes for established medical treatments. Clinicians should tailor dietary recommendations to individual patient characteristics and avoid overstating the therapeutic effects of any single nutrient or dietary pattern.

In summary, dietary interventions, as safe and sustainable lifestyle modifications, may play a role in the prevention, early intervention, and overall care of POI. However, optimal strategies, target populations, and long-term effects of these interventions still need to be defined in high-quality prospective studies and randomized controlled trials. Until stronger evidence becomes available, maintaining a healthy diet, adopting healthy lifestyle habits, and providing individualized counseling remain practical and reasonable approaches in current clinical practice.

## Unresolved issues and perspectives

Although research on the relationship between diet and POI has expanded in recent years, substantial limitations remain with respect to study design, consistency of findings, and clinical translatability. A critical appraisal of current gaps and clarification of future research priorities are essential for advancing this field toward mechanistic understanding and evidence-based clinical application.

### Limitations of observational evidence

Most available studies are cross-sectional or observational in nature, which makes it difficult to determine the direction of the relationship between diet and ovarian function. It remains unclear whether declining ovarian function leads to changes in dietary behavior, whether unfavorable dietary patterns precede ovarian decline, or whether both are influenced by other underlying factors.

Future research should prioritize large-scale, long-term prospective cohort studies incorporating repeated dietary assessments and longitudinal evaluation of ovarian reserve markers. Such studies would help clarify temporal relationships and provide stronger evidence on the role of diet in ovarian aging and POI.

### Phenotypic heterogeneity and insufficient population stratification

The POI is a heterogeneous disorder with diverse underlying causes, including genetic, autoimmune, iatrogenic, and unexplained cases. However, many studies have approached POI as a single disease entity, which may obscure important differences in dietary associations and responses to dietary interventions across etiologic subtypes. Future studies should place greater emphasis on subgroup analyses based on underlying cause and metabolic characteristics to identify women most likely to benefit from nutritional interventions.

### Methodological challenges in dietary assessment

Another major challenge is the accurate assessment of dietary intake. Most studies rely on food frequency questionnaires or short-term dietary recalls, both of which are vulnerable to recall bias and measurement error. Future studies may be strengthened by combining self-reported dietary data with objective nutritional biomarkers and more standardized assessment methods.

### Limited mechanistic insight and insufficient multi-omics integration

Mechanistic understanding of the relationship between diet and ovarian aging remains incomplete. Although diet has been implicated in pathways related to oxidative stress, inflammation, mitochondrial dysfunction, epigenetic changes, and the gut microbiota, much of the current evidence comes from single-pathway studies and experimental models. Closer integration of human observational findings with molecular profiling may help identify the biological mechanisms through which diet influences ovarian aging.

### Scarcity of randomized controlled trials

Randomized controlled trials of dietary interventions in women with POI or diminished ovarian reserve remain scarce, and most have been small or short in duration. In addition, heterogeneity in outcome measures, including AMH, FSH, and menstrual function, limits comparison across studies. More rigorously designed multicenter trials with prespecified endpoints and longer follow-up are needed.

In the longer term, improved phenotyping and molecular profiling may support more individualized nutritional strategies for women at risk of POI.

## Caloric restriction and intermittent fasting in ovarian aging and POI

Beyond dietary composition itself, increasing attention has focused on whether the amount and timing of food intake may influence ovarian aging. Caloric restriction (CR) and intermittent fasting (IF) are two distinct but mechanistically overlapping dietary interventions that demonstrated beneficial effects in preclinical models of reproductive aging, raising interest in their potential relevance to POI.

### Caloric restriction and ovarian reserve: evidence from animal models

Caloric restriction, broadly defined as a sustained reduction in energy intake without malnutrition, is one of the most reproducible interventions for extending lifespan and attenuating aging-related pathology across multiple species. In rodent models, increasing evidence suggests that long-terms CR preserves primordial follicle pool and delays reproductive aging. A recent study in mice demonstrated that 30% CR maintained over an extended period (3–11 months) reduced body mass, improved insulin sensitivity, preserved primordial follicle numbers, and attenuated ovarian macrophage infiltration compared to ad libitum–fed controls (78). Importantly, these effects depended on the duration of CR: short-term CR, whether initiated early or late in the reproductive lifespan, failed to preserve the ovarian reserve, suggesting that sustained rather than short-term energy restriction is required to preserve ovarian reserve (78). Similar findings have recently been reported in nonhuman primates. A 2025 study in rhesus macaques demonstrated that moderate CR over 3 years preserved a more youthful follicular distribution and attenuated age-related ovarian fibrosis—characterized by altered collagen and hyaluronic acid matrices—in aged animals that retained residual cyclicity, suggesting that CR may partially preserve ovarian integrity even at advanced reproductive aging (79).

At the molecular level, CR exerts its ovarian effects through several interconnected pathways. Activation of the SIRT1 (silent information regulator 1) signaling appears to be a key mechanism underlying these effects: CR upregulates the expression of SIRT1, SIRT6, FOXO3a, and NRF1 in ovarian tissue, while simultaneously downregulating p53 and suppressing mTORC1 (mammalian target of rapamycin complex 1) activity (80). These molecular changes suppress premature activation of primordial follicles—a major driver of follicular pool exhaustion—and reduces apoptosis in granulosa cells (80). Consistent with the earlier discussion of the AMPK–PGC-1α axis in mitochondrial biogenesis, CR also enhances mitochondrial function and antioxidant capacity in ovarian cells, thereby reducing oxidative stress induced follicular loss. The suppression of mTOR signaling by CR is mechanistically parallel to the effects of rapamycin, which has independently been shown to preserve the ovarian reserve and improve oocyte quality in aged mice, further supporting mTOR signaling as a potential therapeutic target for delaying reproductive aging (81).

### Intermittent fasting: mechanisms and potential risks

Intermittent fasting encompasses a spectrum of dietary approaches, including alternate-day fasting (ADF), the 5:2 diet, and time-restricted eating (TRE), all of which share the common feature of cyclic periods of caloric restriction or complete fasting. Beyond simple caloric reduction, IF induces a distinct metabolic state characterized by enhanced autophagy—the cellular quality-control mechanism that clears damaged organelles and misfolded proteins—which may not be activated to the same extent during continuous caloric restriction (82). Autophagy plays a critical role in oocyte quality maintenance, and its decline with age has been implicated in the accumulation of dysfunctional mitochondria and impaired oocyte developmental competence (83). By periodically enhancing autophagy, IF may therefore offer a complementary mechanism for preserving the ovarian cellular environment that continuous CR does not fully replicate.

In terms of systemic metabolic effects, IF improves insulin sensitivity, reduce circulating inflammatory markers (including TNF-α and IL-6), lower oxidative stress, and favorably modulate adipokine secretion—all of which are pathways with established relevance to ovarian function as discussed in the preceding sections of this review (84). These systemic changes a metabolic environment that is more favorable for ovarian function, potentially mitigating some of the metabolic and inflammatory insults that contribute to follicular depletion.

However, findings from human studies are less consistent than those from preclinical models, and several limitations should be noted. In healthy women of reproductive age, evidence from human studies remains limited and sometimes inconsistent. Some studies suggest that IF—particularly TRE regimens—may reduce circulating levels of LH, FSH, estradiol, and AMH, raising concerns that intensive fasting regimens may suppress the hypothalamic–pituitary–ovarian (HPO) axis or reduce ovarian reserve markers (84). These hormonal changes may reflect the physiological response to low energy availability (LEA), a state that has independently been recognized as a risk factor for ovarian dysfunction and functional hypothalamic amenorrhea (14). Accordingly, the balance between the metabolic benefits and reproductive risks of IF likely depends on an individual’s metabolic and reproductive status, particularly in women who are lean, physically active, or already at risk of diminished ovarian reserve.

In contrast, IF may be more beneficial in women with metabolic dysfunction. And TRE interventions have been associated with improvements in menstrual regularity, reductions in hyperandrogenism, and improved insulin sensitivity, suggesting that the metabolic rather than the caloric dimension of fasting may be the primary driver of its reproductive benefits in this population (85).

### Clinical translation: current challenges and future directions

Collectively, current evidence suggests that CR and IF may represent biologically plausible approaches for attenuating ovarian aging, particularly through modulation of SIRT1–mTOR signaling, AMPK–PGC-1α–mediated mitochondrial biogenesis, autophagy, and systemic metabolic and inflammatory homeostasis. These pathways converge on the central pathogenic mechanisms of POI discussed throughout this review, supporting further investigation of these interventions in POI.

However, several major barriers remain before these findings can be translated into clinical practice. First, almost all mechanistic data come from rodent models, and the dose, timing, and duration of CR required to achieve reproductive benefits in humans remain undefined. The nonhuman primate data are encouraging but limited to a single study (79), and the optimal timing of intervention—whether CR initiated earlier in reproductive life yields greater benefits—remains an important unresolved question (78). Second, the safety profile of IF in women with known or incipient POI has not been established; the risk of exacerbating hormonal suppression or nutritional deficiencies in an already vulnerable population should be carefully evaluated (84). Third, no randomized controlled trials have specifically evaluated the effects of structured CR or IF protocols on ovarian reserve markers or clinical outcomes in women with POI or diminished ovarian reserve.

Future studies should establish the feasibility and safety of these interventions before large efficacy trial are undertaken. Incorporating measures of autophagic activity and mitochondrial function as mechanistic endpoints may help clarify whether the mechanisms identified in preclinical models are relevant in humans. At present, CR and IF remain experimental approaches for POI prevention and management. Although supported by encouraging preclinical data, their efficacy and safety in women with POI have not yet been established.

## Dietary endocrine-disrupting contaminants and their potential role in POI

In addition to nutrients with potential protective effects on ovarian function, diet is also a major source of exposure to endocrine-disrupting chemicals (EDCs). EDCs are exogenous compounds that interfere with hormonal signaling and disrupt normal endocrine homeostasis (86). Increasing evidence from both epidemiological and experimental studies suggests that dietary exposure to certain EDCs may impair ovarian function, accelerate follicular depletion, and contribute to the development of POI (87, 88).

Among the large number of known EDCs, bisphenol A (BPA) and per- and polyfluoroalkyl substances (PFAS) have attracted particular attention because of their widespread human exposure and growing evidence linking them to ovarian dysfunction. Given the broad scope of this field and space limitations, the present review focuses primarily on BPA and PFAS, which currently have the strongest and most extensively studied associations with ovarian dysfunction and POI (87).

### Bisphenol A and related compounds

Bisphenol A (BPA) is a widely used industrial chemical found in polycarbonate plastics, epoxy resin linings of food and beverage containers, thermal receipt paper, and other food-contact materials. Dietary intake is considered the primary route of human exposure. BPA acts as a xenoestrogen by binding to both classical estrogen receptors (ERα and ERβ) and membrane-associated estrogen receptors, thereby disrupting normal estrogen signaling (86). Epidemiological studies have linked BPA exposure to reduced antral follicle count (AFC) and lower anti-Müllerian hormone (AMH) levels (87). Experimental studies further demonstrate that BPA impairs steroidogenesis, inhibits follicular development, promotes granulosa cell apoptosis, and accelerates primordial follicle activation and depletion (87). BPA exposure has also been associated with epigenetic alterations in ovarian tissue, including changes in DNA methylation patterns that may persist across generations, raising concerns regarding potential transgenerational reproductive effects (88).

### Per- and polyfluoroalkyl substances (PFAS)

The PFAS are a large class of highly stable fluorinated compounds widely used in non-stick cookware, water-resistant food packaging, food processing materials, and industrial applications. Because of their strong chemical stability and environmental persistence, PFAS are often referred to as “forever chemicals.” Human exposure occurs primarily through contaminated food and drinking water, and measurable PFAS levels have been detected in blood, follicular fluid, and ovarian tissue. Epidemiological studies have linked higher circulating levels of several PFAS compounds, including perfluorooctanoic acid (PFOA), perfluorooctane sulfonic acid (PFOS), and perfluorohexane sulfonic acid (PFHxS), with increased risk of POI (89). More recent studies further suggest that next-generation PFAS compounds, including hexafluoropropylene oxide dimer acid (HFPO-DA), may also contribute to ovarian dysfunction (90). Experimental evidence indicates that PFAS exposure induces oxidative stress, disrupts granulosa cell–oocyte communication, impairs steroidogenesis, and promotes ovarian cell apoptosis (89). PFAS have additionally been shown to interfere with epigenetic regulation and hypothalamic reproductive signaling pathways, further supporting their potential role in ovarian aging and POI pathogenesis (91).

To facilitate clinical orientation, the major dietary factors discussed throughout this review—encompassing protective dietary patterns, specific nutrients, emerging dietary strategies, and endocrine-disrupting contaminants—along with their proposed biological mechanisms and clinical implications, are summarized in Table 1.

## Conclusion

Premature ovarian insufficiency (POI) is a complex and heterogeneous disorder with important reproductive, metabolic, and psychosocial consequences. In addition to established genetic, autoimmune, and iatrogenic causes, emerging evidence suggests that diet and nutritional status may also be associated with ovarian aging and susceptibility to POI.

This review summarizes current evidence linking dietary patterns and specific nutrients to ovarian function and outlines several biological pathways through which diet may affect ovarian reserve. These pathways include oxidative stress, mitochondrial dysfunction, inflammation, endocrine and metabolic dysregulation, epigenetic regulation, and interactions along the gut microbiota–ovary axis. Overall, dietary patterns characterized by higher intake of plant-based foods, unsaturated fats, and nutrients with lower inflammatory potential tend to be associated with more favorable indicators of ovarian reserve, whereas Western-style dietary patterns rich in saturated fats, refined carbohydrates, and ultra-processed foods have been linked to poorer ovarian outcomes.

Evidence on individual nutrients, including vitamin D, omega-3 polyunsaturated fatty acids, antioxidants, and other key micronutrients, suggests possible modulatory effects on ovarian function. However, findings remain inconsistent, and the current evidence is not sufficient to draw firm conclusions about causality. Most available data come from observational studies or experimental models, and relatively few well-designed randomized controlled trials have specifically focused on POI or early ovarian dysfunction. At present, dietary interventions should therefore be viewed as supportive rather than primary therapeutic strategies.

Clinically, dietary modification may represent a safe and accessible component of a broader approach to the prevention and management of POI. Attention to overall diet quality, correction of nutritional deficiencies, and individualized dietary counseling may help improve metabolic health, reduce the risk of comorbidities, and enhance quality of life in women at risk of or living with POI.

Further research is needed to clarify temporal relationships, improve dietary exposure assessment, and strengthen mechanistic understanding through better integration of epidemiologic and molecular data. More rigorous longitudinal studies and intervention trials will be essential before specific dietary recommendations can be made with confidence. In the longer term, improved phenotyping and molecular profiling may support more individualized nutritional strategies for women at risk of POI.
